# Increasing medication assisted treatment in rural primary care practice: a qualitative comparative analysis from IT MATTTRs Colorado

**DOI:** 10.3389/fmed.2024.1450672

**Published:** 2024-10-02

**Authors:** Jodi Summers Holtrop, Rebecca Mullen, Kristen Curcija, Claude Rubinson, John M. Westfall, Donald E. Nease, Linda Zittleman

**Affiliations:** ^1^Department of Family Medicine, University of Colorado Anschutz Medical Campus, Aurora, CO, United States; ^2^Department of Social Sciences, University of Houston-Downtown, Houston, TX, United States

**Keywords:** opioid use disorder, rural primary care, qualitative comparative analysis, medication assisted treatment, qualitative research

## Abstract

**Purpose:**

Opioid dependence and use disorders (OUDs) are serious public health crises resulting in a rising number of opioid-related deaths. Medication assisted treatment (MAT), in this case treatment with buprenorphine, is an evidence-based solution to combatting OUD; however, MAT has been largely unavailable in rural areas. This study investigated what it took to increase MAT in rural Colorado primary care practices.

**Methods:**

Mixed methods study using qualitative and quantitative data collected from interviews, observations, surveys, and practice-reported data. Participants were staff members from 35 rural primary care practices in Colorado, United States. We qualitatively analyzed the data, then transformed the data, then analyzed it using qualitative comparative analysis (QCA).

**Results:**

Having a MAT waivered prescribing clinician on staff and a MAT system in place were necessary conditions to providing MAT (consistency = 1.0; coverage = 0.53 & 0.39 respectively). Practice size (number of providers) was associated with differences in conditions that provided sufficient aspects for MAT provision. Small (1–2 medical providers), non-private practices benefited from the presence of behavioral health and a clinician with MAT experience. Medium sized practices (3–5 providers) whether private or not benefited from behavioral health, often in combination with a clinician with MAT experience. In large practices (6 or more providers), behavioral health was not a factor while having a clinician with MAT experience mattered half of the time.

**Conclusion:**

Implementation of MAT in rural primary care is a complex task that may benefit from the resources of behavioral health and a clinician with prior MAT experience.

## Introduction

The opioid epidemic is a major public health problem in the United States (1, 2). Treatment for OUD with buprenorphine (a partial opioid agonist at the mu-opiate receptor), naltrexone (an opioid antagonist), or methadone (a synthetic opioid that acts as a full agonist), frequently referred to as medication assisted treatment (MAT), is an effective, evidence-based approach (3, 4). However, only 25% of U.S. adults with OUD receive MAT (5).

Efforts to close this treatment gap, save lives, and improve the quality of life for individuals and communities include engaging primary care, where Americans receive about half of all their health care (6, 7). Primary care practice has been considered a potential mitigator of OUD with their ability to screen, diagnose, and treat patients by a single clinical team (8). MAT can occur in outpatient settings (9) and can leverage existing chronic disease management models embraced by primary care. Prior to 2023, a primary care clinician was required to obtain a DEA waiver to prescribe buprenorphine to treat opioid use disorder. Obtaining the waiver required 8 h of approved training. Once trained, the DEA offered an “X” waiver that allowed the primary care clinician to prescribe buprenorphine for opioid use disorder. More recently, to increase access to buprenorphine, this waiver has been eliminated so that all clinicians with a DEA license can prescribe buprenorphine for opioid use disorder. Unfortunately, the implementation of MAT in primary care has been slow, with barriers including misconceptions about the patients needing treatment, stigma, and lack of trained staff (10–13). While the Consolidated Appropriations Act of 2023 eliminated the federal requirement for clinicians to have a special waiver to prescribe buprenorphine for OUD (14), this waiver requirement previously acted as another barrier to MAT implementation (11, 15, 16). Following the trend in other states, only 54% of clinicians in Colorado who had a DEA waiver wrote a buprenorphine prescription that was filled in 2016 and 2017. Further, only 8% of waivered clinicians were located in rural Colorado counties, although rural counties experience the highest rates of drug overdose deaths (6, 17). The literature is silent on why exactly this is the case but this may be potentially due to the overall shortage of medical and behavioral health clinicians in rural areas.

Implementing Technology and Medication Assisted Treatment Team Training in Rural Colorado (IT MATTTRs) is a partnership of researchers, community members, and MAT experts convened to address the OUD crisis in rural Colorado. In Colorado 12 counties have overdose death rates of more than 20 per 100,000 and are among the highest in the nation; seven of these counties are located in the region where IT MATTTRs was implemented (18). IT MATTTRs is a practice and community intervention to improve knowledge and awareness of OUD and increase access to buprenorphine treatment for OUD in rural Colorado primary care practices. Started in 2016 when waivers were still required for OUD treatment with buprenorphine, IT MATTTRs included an evidence-based practice team training on OUD treatment with buprenorphine tailored to rural primary care practices (19–23). Although naltrexone and methadone are also evidence-based MAT, this project focused on buprenorphine because it can be prescribed in primary care. Administering methadone must be done at a Certified Opioid Treatment Program, which are not accessible in our rural study region, and the long withdrawal period of naltrexone was not appealing to clinicians participating in the IT MATTTRs study. For these reasons, this study supported the use of treatment with buprenorphine.

Recognizing the challenges and complexity of organizational change in primary care practices (24), we used Qualitative Comparison Analysis (QCA), a novel analytic method, to conduct a rigorous exploration of the key factors needed to increase the provision of MAT to patients in practices receiving the IT MATTTRs Team Training. This paper describes the results of QCA based on our qualitative and other data collected. Our results build on reports of barriers and contribute to detailed understanding of successful implementation of MAT in rural primary care practices.

## Methods

We obtained approval from the Colorado Multiple Institutional Review Board to conduct this work. This project was supported by grant number R18HS025065 from the Agency for Healthcare Research and Quality (AHRQ).

### IT MATTTRs interventions

The IT MATTTRs study included community-focused interventions (22) and opportunities for clinicians (physicians and advance practice providers) to complete training and obtain the previously-required DEA waiver to prescribe buprenorphine for OUD. The program also provided training to practice teams in rural Colorado to enhance understanding of OUD and OUD treatment and facilitate the implementation of MAT. The training covered the epidemiology of OUD, pharmacology of buprenorphine, neurobiology of addiction, and detailed MAT steps. Forty-two practices were randomly assigned to receive training via the in-person Shared Onsite kNowledge Dissemination (SOuND) Team Training™ model (n = 24) or an Extension for Community Health Outcomes (ECHO) tele-health model (n = 18). Described in detail elsewhere (20), the training was offered to all staff and clinicians at participating practices.

### Setting and participants

Forty-two out of approximately 80 total primary care practices from two regions of Colorado, the eastern plains area (served by the High Plains Research Network, or HPRN) and the San Luis Valley (served by the Colorado Research Network, or CaReNet), participated in the IT MATTTRs study and received the IT MATTTRs Practice Team Training. All practices were in a 24-county region located entirely in rural areas, and 13 of the counties have frontier designation (fewer than 6 people per square mile); combined, this study region geographically covers 37% of the state of Colorado. All counties are designated Mental Health Professional Shortage Areas (25). See Table 1 for a list of available primary and behavioral health resources in study region.

**Table 1:** Primary and behavioral health resources in study region.

Description	Total #
Hospitals	20
Primary care practices	86
MD/DO	121
Physician assistants	43
Nurse practitioners	38
Licensed behavioral health care providers	53
Certified addiction counselors	21
Certified primary care buprenorphine prescribers	2
Certified psychiatrist buprenorphine prescribers	2

Participants in this evaluation included a variety of practice member roles including at least one medical provider (physician, nurse practitioner or physician assistant), one clinical staff member (usually a medical assistant or clinical supervisor) and, if possible, other roles such as behavioral health provider, practice manager, or health educator/care coordinator. Baseline interviews were conducted in 42 practices and follow-up interviews were completed in 35. Two to five individuals were interviewed per practice, depending on the practice size. The IT MATTTRs Practice Team Training reached 441 team members across all practices and roles (out of an eligible 676 individuals) and a total of 108 interviews were completed across all practices. While all members of the participating practice teams were encouraged to attend the team training, some individuals missed the training due to scheduling obligations or staff turnover; however, all interviewees were familiar with the IT MATTTRs study and their practice’s involvement in it.

### Data collection and instruments

The data for this evaluation was collected from a variety of IT MATTTRs data sources including baseline observations, MAT Implementation Checklist at baseline, 6 and 12 months post-intervention, and interviews at baseline and 6 months post-intervention. We conducted observations at each practice at baseline (pre-intervention), which captured information such as the physical layout and space, medical record procedure, work teams, and practice atmosphere. The MAT Implementation Checklist was completed by either a clinician or practice manager and measured the level of MAT implementation using a 26-item checklist, at baseline, and 6 and 12 months after the practice team training. Demographic data were collected from individual practice team members who participated in the qualitative interviews using a short survey (age, gender, race/ethnicity and length of time in the profession and at the practice).

We developed a semi-structured interview guide to determine practice member perceptions and current practices regarding opioid use, OUD, and OUD treatment. The baseline interview guide covered the practice member role, current practices and protocols in managing patients with both regular opioid use and OUD, and goals, concerns and intentions regarding providing MAT to patients in their practice. Follow-up interviews (held after intervention completion) included the same topics as well as practices’ experiences with the intervention and their current procedures and future plans regarding MAT. Interviews lasted 30–60 min each and were conducted by a study team member either in person or by telephone. All interviews were conducted either by a doctorally-trained qualitative researcher or other study team members trained and supervised by that qualitative researcher. Study team members practiced with interviews until they demonstrated competency. Participants were compensated with a gift card. A memo form was completed immediately after each interview to capture key summary points and reflections. Interviews were audio recorded, transcribed, and loaded into ATLAS.ti version 8 (ATLAS.ti GmbH, Berlin, Germany). Baseline interviews and observations were conducted between July, 2017, and September, 2018, and results are reported elsewhere (21). Follow-up interviews were conducted between November, 2018, and January, 2020.

### Analysis

#### Approach

For analyzing the baseline data, we used a grounded hermeneutic editing approach (26). This approach is similar to a grounded theory approach but includes editing as an “organizing style of analysis that helps bring forth greater understanding of or meaning from text or data” (26). For the qualitative analysis considering both the baseline and follow-up interview data and other sources of data, we used a variation of a rapid qualitative analysis approach (27–29).

#### Data sources and practice template creation

In this process, we created a summary template for each practice (see Supplementary material S1). The template covered: practice information, OUD and MAT experience, resources including behavioral health, opioid prescribing procedures, and evidence of diversion (i.e., if interview participants described examples of diversion, or sharing medications with others not prescribed), or inherited prescribing (i.e., assuming opioid prescriptions for inherited patients) as captured in the interviews. An qualitative core team member completed the template after reviewing all the data sources for each practice. The data sources included all the baseline and follow-up interview transcripts, the observation field notes, the fidelity checklist (for MAT provision or referral system), and surveys (for practice demographics).

Next, another core team member checked over the completed template using the same data sources. Any discrepancies were discussed and rectified, which sometimes required revisiting the data and considering new categories on the template for summarization. After a summary template was completed for each practice, overall summaries across practices were considered.

#### Summary template creation

Then the team discussed the convergence of these summaries noting areas of overlaps and divergence. The team considered the meaning and relevance of results emerging from the data. These data were presented to the larger research team for discussion and feedback, then refined and finalized for consistency and implementation considerations. These efforts allowed the core team to revisit the data, confirm, modify, or refute initial impressions, and identify additional areas for further analysis. Throughout the entire analysis process, the lead qualitative analyst kept a summary document as an audit trail to record the discussion elements and decisions made. The completed summary templates provided the essential information for further analysis.

#### Explanatory condition identification

Next the analysis team reviewed all the data sources and identified the factors most likely to be related to the presence of the outcome of interest, providing MAT. Potential explanatory conditions were identified based on clinical expertise, literature review, and feedback from the larger IT MATTTRs team. Once the potential explanatory conditions were identified, the analysis team organized the data into an Excel matrix, with the rows of the matrix enumerating the practices and the columns enumerating the explanatory conditions Supplementary material S1. The explanatory conditions included those emergent from the qualitative analysis (such as leadership buy-in), important practice descriptive health system information (such as practice size), and other factors (such as having a behavioral health provider). The qualitative team then reviewed the matrix in multiple passes to highlight and group categories consisting of similar dimensions; these were then reviewed and approved by the larger study team. Corroborating/legitimating occurred through review of existing literature and seeking out of associated experiences to confirm or refute insights from the analysis. After initial analysis identified data to support one theme or interpretation, particular effort was devoted to finding negative or disconfirming evidence, essentially examining the data and looking for alternative explanations or findings.

### Qualitative comparative analysis

Given the nature of the data appearing to potentially be configurational, the research team decided to use qualitative comparative analysis (QCA) (30–32), an analytical method used to study complex causality and the effects of context. QCA is uniquely useful in determining what combination of factors may contribute to a particular outcome. It may be used to analyze a mix of quantitatively-and qualitatively-derived data and, in contrast to traditional statistical analysis, is not dependent upon random or representative sampling. Based on Boolean algebra, which deals with logical operations (33), QCA is not constrained by degrees-of-freedom restrictions in the way that conventional statistics are and may therefore be applied to samples of arbitrary size (32, 34). To complete the QCA, we used the protocol outlined by Rihoux and Ragin (30). We first reviewed the emergent themes identified in the analysis above, arriving on an initial set of likely explanatory variables (“conditions” in the terminology of QCA). We then rescaled (“calibrated”) these conditions onto a 0.0 to 1.0 scale for each practice. Table 2 presents the conditions and the guide the team used in coding them. The outcome condition was having a formal MAT system, defined as a system for identification of patients suffering OUD along with referral or treatment, behavioral health support/referral, peer support or referral, and follow-up plans. The scores represent the degree to which the practice exhibits the specified condition. The members of the analysis team then independently scored each practice using the coding guide, before coming together to compare and resolve any discrepancies. The final scoring involved going back to the transcripts, recalibrating if necessary, and determining each practice’s final scores. This process was repeated for the outcome of providing MAT. The analyses were conducted using Kirq,^1^ a publicly available software package for QCA developed by co-author CR.

**Table 2:** Outcome and explanatory conditions.

Condition	Description of condition	Calibration	Rationale
Providing MAT	Extent to which the practice has at least one medical provider providing MAT to patients at that clinic	1 = providing MAT fully with systems in place 0.8 = generally providing but is still bumpy, erratic 0.6 = quietly providing MAT but not much or not advertised 0.2 = did a few and stopped 0 = not providing MAT	Outcome of interest
Practice Size	Small practice = 1–2 medical providers; Medium = 3–5; Large = 6 or more	N/A. Practice size was used as a contextual condition with separate QCA analyses for small, medium, and large practices	Size may have an effect on ability or interest in providing MAT
Private practice	Practice is owned privately by the physician or medical provider or those in the practice	1 = private practice 0 = practice is owned by a hospital	This practice structure may operate differently than others
Clinician with MAT Experience (Clinician)	At least one medical provider (with prescribing authority like a physician or APP) has had exposure to or themselves provided MAT before either at this practice or elsewhere	1 = had experience providing MAT themselves 0.8 = has experience with observing MAT being provided in own practice 0.6 = has had some exposure in their training but has not provided it themselves or in own practice 0 = has not experience or exposure to MAT	Those who have already provided MAT makes for 1) mental switch of being willing to do it and 2) hurdle of having done it already so easier on-ramp to doing again; exposure maybe more willing to consider doing it Some practices might have a MAT prescriber with experience (or a waiver) who is choosing NOT to prescribe
MAT prescriber on staff (Prescriber)	Practice has a clinician with a DEA waiver	1 = Clinician with DEA waiver to prescribe MAT at practice 0 = No clinician with DEA waiver to prescribe MAT at practice	Some practices might have a MAT prescriber with experience (or a waiver) who is choosing NOT to prescribe
Behavioral Health in the Practice	Having at least a part-time person physically located in the practice that provides mental and behavioral health support	1 = BH in the practice at least part-time regardless of employment and has a least some capacity to take on MAT patients 0.6 = so occasional and already overbooked that no capacity to take on MAT or very occasional BH in the practice and not familiar with MAT 0 = no BH in the practice even if have referral source	Since many perceive BH support as a needed component to providing MAT, this may make a difference in willingness to do it
Leadership Buy-in for MAT	The extent to which leadership at the practice and health system level (if applicable) is supportive of providing MAT	1 = strong leadership support across range of leaders with emphasis on major decision maker(s) 0.6–0.9 = range of generally having support 0.1–0.4 – range of generally not supportive 0 = no leadership support or blocking efforts to do MAT	When leadership is not in support, then the practice often cannot move ahead with providing MAT
Practice Champion or Key Person	The presence of a person who was in charge and made things happen for the MAT efforts; usually person with passion, but does not have to be	1 = had champion <0.5 = not strong champion—someone voluntold to do it/no passion/capacity 0 = no champion	If there is someone there whose job it is to get MAT going, this could influence progress on making it happen
MAT system in place	Extent to which the practice has some way of having their patients get to MAT help. A formal MAT system included identification of patients suffering OUD along with referral or treatment, behavioral health support/referral, peer support or referral, and follow-up plans	1 = providing MAT directly or have strong and consistent referral partners that their patients get referred to (including follow-up) 0.1–0.45 = range of having some referrals but not consistent or strong or not MAT-specific 0 = neither in place	If they have a way to refer to or provide MAT, this affects the outcome of getting patients to MAT

The QCA revealed that a number of conditions hypothesized to increase the provision of MAT were not causally explanatory. Such conditions were both contextual (e.g., is the practice part of a health system network; is the practice designated as a federally qualified health center) and proximate (e.g., does the practice serve a large number of patients with OUD; is MAT already provided in the community). Table 2 lists the conditions that were included in the final analysis; Supplementary Table 1 lists those conditions that were examined but found to be causally unimportant. One condition found to be of particular importance was practice size; the QCA revealed different causal “recipes,” or critical combinations of factors, for providing MAT depending upon whether the practice was small (1–2 medical providers), medium (3–5 medical providers), or large (6+ medical providers). The decision was therefore made to split the data set into three and conduct separate QCAs for the different practice sizes.

As described by Ragin (32, 34), QCA seeks to identify necessary and sufficient conditions that are related to the presence of an outcome. A unique benefit of QCA is that it is sensitive to issues of causal complexity (that multiple conditions may operate in tandem) and equifinality (that there may be multiple pathways or “recipes” to realizing an outcome). A necessary condition is a condition (or combination of conditions) that must be present for the outcome to occur; its absence prevents the outcome from occurring. A sufficient condition is a condition (or combination of conditions) that, when present, ensures that the outcome will occur. Necessity and sufficiency can be imperfect: it is perfectly appropriate to identify a particular combination of conditions as “almost always” necessary or sufficient.

The degree to which a given condition or combination of conditions is necessary and/or sufficient for the outcome is measured by two goodness-of-fit metrics: consistency and coverage. Ranging between 0.0 and 1.0, consistency measures the strength of the necessary or sufficient condition. Established standards for QCA use thresholds of > = 0.9 to establish necessity and > =0.8 to establish sufficiency. Coverage also ranges between 0.0 and 1.0 and measures empirical prominence; recipes with higher coverage scores explain a greater fraction of the outcome than recipes with lower coverage scores. A low coverage score may or may not indicate that the necessary or sufficient condition is trivial. There are not standard coverage scores for establishing necessity or sufficiency: a condition that explains only a small fraction of cases may still be substantively important.

To conduct a sufficiency analysis using QCA, the raw data matrix is first reformulated as a “truth table.” Presented in Table 3, a truth table lists every possible combination of conditions, the number of practices belonging to each truth table row, and the associated consistency score, which expresses the degree to which the cases associated with a particular truth table row exhibit the outcome. Those rows lacking cases (referred to as “remainders”) are omitted to save space.

**Table 3:** Truth tables describing large-, medium-, and small-sized practices providing MAT, sorted by consistency (consistency threshold: 0.80; proportion threshold: 1.0; frequency threshold: 1) remainders are excluded.

Private Practice	Behavioral Health	Clinician w/ Experience	Prescriber in Practice	Buy-in	Key Person	MAT System	N	Consistency	Outcome
*Truth table 1: large practices*									
False	True	True	True	True	True	True	1	1.00	True
False	False	True	True	True	True	True	1	1.00	True
False	False	False	True	True	True	True	1	0.87	True
False	True	False	True	True	True	True	3	0.21	False
False	False	False	False	False	False	True	1	0.00	False
*Truth table 2: medium-sized practices*									
True	True	True	True	True	True	True	1	1.00	True
False	True	True	True	True	False	True	1	1.00	True
False	True	False	True	True	True	True	1	1.00	True
False	True	True	True	True	True	True	1	0.92	True
True	False	False	False	True	False	True	1	0.00	False
False	True	False	False	True	False	True	1	0.00	False
False	False	False	True	False	False	False	1	0.00	False
False	False	False	False	False	False	False	1	0.00	False
*Truth table 3: small practices*									
False	True	True	True	True	True	True	2	0.92	True
True	False	False	True	True	True	True	1	0.90	True
False	True	True	True	False	True	True	1	0.33	False
False	True	False	True	False	True	True	2	0.33	False
True	False	True	True	True	False	True	1	0.00	False
False	True	False	True	True	False	True	1	0.00	False
False	True	False	False	True	True	True	1	0.00	False
False	True	False	False	True	False	True	1	0.00	False
False	True	False	False	True	False	False	1	0.00	False
False	True	False	False	False	False	True	1	0.00	False
False	False	False	True	False	False	True	1	0.00	False
False	False	False	False	False	False	True	3	0.00	False
False	False	False	False	False	False	False	4	0.00	False

After constructing the truth tables, software is used to perform a minimization process that identifies and eliminates logically-irrelevant conditions and mathematically reduces the truth table to a set of Boolean expressions.

## Results

Some practices were unable to complete follow-up interviews due to practices closing, “shared” clinical staff across multiple participating practice sites, and a small number of practices that chose not to participate in follow-up interviews. We did not include data from practices that did not participate in follow-up interviews, which resulted in 35 practices out of 42 total with complete information. Descriptive information about the participating practices and practice members are shown in Table 4.

**Table 4:** IT MATTTRs practice interview participant descriptions.

Demographic factor	*N* = 108	
	*n*	%
*Gender*		
Male	18	17%
Female	90	83%
*Age*		
Age 18–35	29	27%
Age 36–64	73	68%
Age 65+	5	5%
Missing	1	1%
*Race*		
White	92	85%
Black/African American	2	2%
Asian	1	1%
American Indian/Alaska Native	2	2%
Native Hawaiian or Other Pacific Islander	0	0%
Other	15	14%
Missing	0	0%
*Hispanic or Latino*		
Yes	38	35%
No	70	65%
*Practice role*		
Clinician	38	35%
Nurse care team (RN, LPN, MA)	33	31%
Administrative (manager, reception, med records, etc.)	33	31%
Behavioral health provider	1	1%
Care manager	9	8%
Missing	1	1%

### Necessity results

The necessity analysis revealed two conditions common to all practices that were providing MAT within 6 months of the conclusion of the study (Table 5). All such practices had a MAT prescriber on staff and MAT system in place, and both conditions exhibited perfect consistency with a consistency score of 1.0. The latter condition is, by definition, trivially necessary for delivering MAT, which is reflected in the relatively low coverage score of 0.39. The relatively higher coverage score of 0.53 for the former condition indicates that while all practices prescribing MAT also have an MAT prescriber on staff, there are also many practices with an MAT prescriber on staff that are not providing MAT.

**Table 5:** Necessary conditions for providing MAT (consistency threshold: 1.0).

Term	Consistency	Coverage
Prescribing provider	1.00	0.53
MAT system in place	1.00	0.39

### Sufficiency results

**Figure 1:**
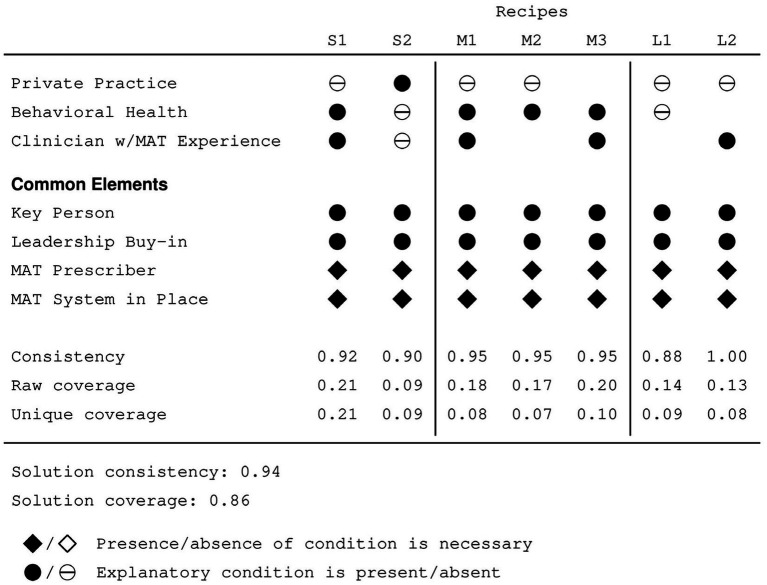
Fiss chart describing characteristics of (S)mall, (M)edium, and (L)arge practices providing MAT.

The sufficiency analysis revealed that practice size was a crucial explanatory condition, with seven different recipes associated with three different practice sizes—two recipes describing small practices, three describing medium practices and two describing large practices. These results are reported in Figure 1 (35), which presents a Fiss configuration chart of seven recipes. In a Fiss chart, the recipes are read vertically, with circles indicating sufficient conditions and diamonds indicating necessary conditions. A filled glyph indicates the presence of a condition and a crossed glyph, its absence. When no glyph is printed, it indicates that the QCA minimization process has identified that condition as irrelevant within the context of the recipe: some practices exhibited the condition while others did not, yet all exhibited the outcome. Overall solution consistency is 0.94, indicating that a practice described by one (or more) of these recipes almost always was providing MAT at 6 months. Overall solution coverage is 0.86, indicating that most (but not all) practices that were providing MAT at 6 months are described by one or more of these recipes.

Across the three practice sizes, all seven recipes share four conditions, indicating that these conditions that their relevance is independent of practice size. In addition to the two necessary conditions identified by the necessity analysis, each recipe also includes the presence of leadership buy-in and a key person driving the MAT program. Note that the fact that these two conditions are common to all seven recipes does not imply that they are necessary for providing MAT. Recall that solution coverage is less than 1.0, which indicates that there are practices providing MAT that are not explained by these recipes; that is, while these two conditions are certainly important to understanding the provision of MAT, the results of the QCA indicate that other pathways to providing MAT are also possible that lack one or both of these conditions.

Among small practices, an important condition was whether the practice was or was not private. Small private practices providing MAT lacked both a behavioral health practitioner as well as a clinician with previous experience providing MAT. In contrast, small non-private practices had staff members fulfilling both roles. Whether the practices were private or not does not appear to be particularly crucial for medium sized practices; some were and some were not. However, all the recipes describing medium-sized practices included the presence of a behavioral health provider; two also included a clinician with previous MAT experience. Two recipes describe large practices, none of which were private. One recipe describes practices that lacked a behavioral health practitioner and may or may not have a clinician with previous MAT experience. The other recipe describes practices that possess a clinician with previous MAT experience and may or may not have a behavioral health practitioner.

## Discussion

This analysis identified practice characteristics that are present in study practices that participated in a team-based training and were providing MAT. While there were numerous contemporary statewide initiatives to increase OUD treatment with buprenorphine, the communities participating in IT MATTTRs had a significantly greater increase in treatment than the rest of the state, as we previously reported (23). The results of the present study help inform practice-focused strategies to engage primary care in treating OUD with buprenorphine.

Surprisingly, having a MAT prescriber in place was not present across all configurations, and not all practices with a waivered prescriber were engaged in the delivery of MAT. The results of this evaluation indicate that having a waivered buprenorphine prescriber is necessary but not sufficient to provide MAT in primary care practices. Having a key person or champion as well as leadership buy-in surfaced as important elements to MAT provision.

Differences in configurations tended to split out based on practice size, which may represent a proxy for resources. Interestingly, among small, private practices, neither behavioral health nor a clinician with prior MAT experience were relevant. This may reflect the flexibility of small private practices with clinicians who seek innovations and resources that keep their practice up-to-date and do not need to seek administrative approval to implement innovations. For medium and larger sized practices, the differences in configurations are less striking, with only one of the five configurations demonstrating no behavioral health presence.

The QCA identified many conditions as irrelevant that were initially expected to matter. Contextual factors like strong perceived community need and lack of a local MAT provider did not appear to be important factors in explaining MAT provision. Nor were health system or hospital affiliation. Similarly, practice change capacity and receiving either the SOuND or ECHO-delivered team training were also not conditions that entered into any configuration. The IT MATTTRs Team Training aims to engage full practice teams, strengthen leadership understanding of the importance and feasibility of MAT in primary care, and identify and activate practice champions, based on findings from numerous studies examining primary care practice improvement. One possibility is that Team Training serves to support these conditions versus standing as its own condition. Further, the comprehensive training may have made other conditions less relevant or necessary for increasing MAT by providing substantial background on the need for MAT in primary care as well as the support required to implement, when the key necessary and sufficient conditions were present.

While recent trends in prescribing behavior indicate increasing prescriptions written by primary care clinicians compared to prescribers in other specialties (36–38), continued efforts to provide MAT in primary care are crucial to expanding access to treatment for OUD. The Consolidated Appropriations Act of 2023 expanded prescribing authority by eliminating the federal requirements of a waiver to prescribe buprenorphine (14), discipline restrictions, and patient limits. Additional types of professionals such as different levels of substance abuse counselors or psychologists may enhance service provision, however, hiring such professionals in rural areas can be challenging. Additional opportunities for increasing MAT implementation efforts may come from understanding the characteristics of current buprenorphine prescribers. The original Agency for Healthcare and Quality grantees reported several facilitators and barriers to MAT implementation in primary care (19). They report that practice culture related to opioid use stigma and community knowledge of treatment options are important factors in uptake of MAT in primary care. Secular changes in opioid use and abuse culture, with decreased opioid prescribing and increased availability of fentanyl analogs has impacted the opportunity to identify potential patients in primary care practices (39–41).

### Limitations

Qualitative work inherently is not meant to be inferential; it is intended to provide insight into what the selected group of participants might be experiencing or perceiving. Thus, this population of practices from selected rural Colorado may not be applicable to other rural populations. Even though we had high participation, it is possible that some selection bias may have occurred such that the sample was predisposed toward implementation of MAT. Multiple methods were used to validate the results including triangulation across researchers and multiple analysis methods; however, the results may have bias or misinterpretation of the stories shared in the interviews or through other methods. This study occurred before the COVID-19 pandemic, and COVID has had a substantial impact on OUD, overdose deaths, and how OUD treatment is provided. This study does not address that concern.

## Conclusion

IT MATTTRS was a multi-component intervention for rural Colorado that included practice team training, prescriber waiver training, and community education. Overall, IT MATTTRs resulted in an increase in the number of primary care practices providing MAT through buprenorphine. The QCA results found that, as expected, having a waivered prescriber was necessary prior to the Consolidated Appropriations Act of 2023. Furthermore, the analysis revealed that, while important, prior MAT experience and behavioral health support were not required elements in all cases. In our data, successful implementation of buprenorphine treatment for OUD also entailed the presence of a practice champion and support from practice leadership. However, the QCA did not identify these as necessary conditions, indicating that they may not in fact always be required. Future research should investigate whether buprenorphine treatment for OUD can be successful in their absence and, if so, under which contexts. The results further revealed important differences by practice size, which may help inform practice-focused strategies to engage primary care in treating OUD with buprenorphine.

## Data Availability

The raw data supporting the conclusions of this article will be made available by the authors, without undue reservation.

## Footnotes

^1^ http://grundrisse.org/qca/download
