# Supplementary material for: Increasing medication assisted treatment in rural primary care practice: a qualitative comparative analysis from IT MATTTRs Colorado
Source: Front Med (Lausanne). 2024 Oct 2;11:1450672. doi: 10.3389/fmed.2024.1450672 (PMC11479968; doi:10.3389/fmed.2024.1450672)
Supplement: Supplementary file 1 [file Table_1.docx]

Supplementary Digital Content

Table 1. **Conditions found to be causally unimportant for explaining MAT uptake**

| **Condition** | **Description of Condition** | **Calibration** | **Rationale** |
| --- | --- | --- | --- |
| In Health System | Practice is part of a larger network of health care entities | 1 = part of a practice network or owned by a hospital  0 = independent practice not connected with other practices or health care entities | Health systems may have more resources to take up MAT; independent practice may be more nimble |
| Community Mental Health Center | Practice is a CMHC | 1 = community mental health center  0 = not a community mental health center | This practice structure may operate differently than others |
| FQHC | Practice is designated as a federally qualified health center | 1 = FQHC | This practice structure may operate differently than others |
| Payment for MAT a concern | Uncertainty about how they would get paid and if enough payment | 1 = how to bill and code and get paid for providing MAT is a concern  0 = not mentioned or not a concern | May be an implementation challenge if no funding mechanism |
| Large Number of Patients with OUD in the practice | Perception by practice staff members that many patients in the practice would benefit from having MAT | 1 = practice perceives that many patients are suffering from OUD and would benefit from MAT  Use range if needed with range for degree of number perception  .6-.8 = have some patients who could benefit, but not a large number  .2-.4 = imply that they don’t have many patients that could benefit  0 = practice perceives that no or almost no patients in the current practice would need MAT | If there are a lot of patients in the practice with this need and it is perceived that these patients would participate and benefit, perhaps the practice will be more likely to want to provide MAT themselves for these patients |
| No accessible/ useful local MAT provider | MAT is not provided in the community or the MAT provider is not working in some way (such that referral does not work) | 1 = MAT not available/ accessible in the community  .7-.9 = basically not available because of severe issue using the MAT provider  .1-.4 = generally available, but some mild issues with use/referral, maybe a bit too long of distance but do-able  0 = MAT available locally | If available locally, may not need to do it within the practice |
| Strong Perceived Community Need in that local community for MAT paired with practice perception of role to serve the community | The perceived need of the practice members of opioid use in the community with mental model of role of the practice is to serve community needs | 1 = strong community need and need to serve community (our role as the practice to do this)  .8 = strong need but unsure if it is their role  .2 = very little interest or perceived need in serving community in this way  0 = no community need for MAT and/or not feeling their role is to serve the community in this way | If there is a need in the community, perhaps the practice feels they need to step-up and provide help in this area |
| Practice Change Capacity | The ability of the practice to effectively make changes to institute new services – not MAT just in general | 1 = previous experience with successful practice change  0 = no practice change capacity evident | Even if a practice wants to do something, they still need the ability to do it |
| Good experience with the practice staff training | Practice members felt that the training they received was a positive experience (i.e. training model, content appropriate, no issues) | 1 = Good experience with training; really activated effort  0 = some aspect of this not being a good experience | Frustration with the training or low uptake may thwart efforts to move forward effectively |
| Training | Is ECHO or SOuND offered at the practice site? | 1 = IT MATTTRsPractice Team Training through ECHO Colorado  0 = IT MATTTRsPractice Team Training through SOuND Team Training | Type of training may make a difference in knowledge uptake and eventual use of MAT |
